# The proteomic landscape of *Toxoplasma gondii* extracellular vesicles across diverse host cell types

**DOI:** 10.3389/fcimb.2025.1565684

**Published:** 2025-03-18

**Authors:** Teresa Cruz-Bustos, Anna Sophia Feix, Karin Hummel, Sarah Schlosser, Ebrahim Razzazi-Fazeli, Anja Joachim

**Affiliations:** ^1^ Institute of Parasitology, Department of Biological Sciences and Pathobiology, University of Veterinary Medicine Vienna, Vienna, Austria; ^2^ VetCore Facility (Proteomics), University of Veterinary Medicine Vienna, Vienna, Austria

**Keywords:** *Toxoplasma gondii*, exovesicles, microvesicles, proteome, mass-spectrometry

## Abstract

**Introduction:**

Extracellular vesicles (EVs) are emerging as powerful tools used by pathogens to manipulate host cells, delivering molecular cargo that rewires cellular processes and the immune response. *Toxoplasma gondii*, a globally distributed parasite capable of infecting nearly all nucleated animal cells, uses this strategy to thrive in diverse host species and tissue environments.

**Methods:**

Here, we reveal the adaptability of *T. gondii* EVs through proteomic analysis of vesicles released from tachyzoites cultured in four different host cell types: human fibroblasts, green monkey kidney epithelial cells, mouse myoblasts and porcine intestinal epithelial cells.

**Results:**

A core set of 1,244 proteins was consistently identified across TgEVs, defining a conserved signature. Beyond this conserved cargo, host-cell specific variation revealed how *T. gondii* fine-tunes EV content to exploit different cellular environments. Functional enrichment analyses revealed roles for TgEVs in targeting host protein synthesis and stress response pathways, with implications for immune evasion and infection spread.

**Discussion:**

These findings provide insight into the potential role of EVs in host-pathogen interactions and help us understand the adaptive strategies used by *T. gondii* to survive and spread.

## 1 Introduction

Extracellular vesicles (EVs), a heterogeneous group of particles that are released by cells, are delimited by a lipid bilayer and have a crucial role in intercellular communication in all living organisms. They are broadly classified into small and large EVs, distinguished by their size, biogenesis and molecular composition (Welsh et al., 2024). Small EVs, which are smaller than 200 nm in diameter and of endocytic origin, are released following the fusion of multivesicular bodies with the plasma membrane and facilitate the transfer of molecular signals. Large EVs are characterized by a more heterogeneous shape and size, ranging from 0.2 to 1 μm in diameter, and are shed directly from the plasma membrane (Welsh et al., 2024). These EVs are instrumental in the transfer of a diverse array of molecular signals, including proteins, lipids, mRNA, micro-RNA (miRNA) and other small non-coding RNA species, between cells, significantly impacting the phenotype and function of recipient cells. EVs have a pivotal role in modulating both physiological and pathological processes (Colombo et al., 2014). Transmitting signals in EVs eliminates the need for cell-cell contact and allows cells to deliver messages to remote sites (Marcilla et al., 2014).

In parasitology, EVs have gained prominence for their role in modulating host-pathogen interactions, although studies on parasite-derived EVs are still limited. In protozoan parasites, EVs can be directly released from the organellar compartments of the parasite or via host cells that are either infected by the parasite or stimulated by antigens, as a response to physiological stressors in both *in vitro* and *in vivo* environments (Olajide and Cai, 2020). Extracellular vesicles have multifaceted roles in parasite biology, including cell-cell communication, transfer of proteins, lipids and nucleic acids, and transport of bio-reactive macromolecules. They are instrumental in enhancing host-parasite interactions, modulating the host immune system and inducing inflammatory responses (de Souza and Barrias, 2020). Specifically, EVs secreted by protozoans such as *Toxoplasma gondii*, deliver virulence factors and other parasite-derived molecules to host cells (Feix et al., 2024), enabling the parasite to manipulate the host environment for its own benefit. *Toxoplasma gondii* have developed sophisticated mechanisms to manipulate the host cellular environment to their advantage (Quiarim et al., 2021). This communication relies on EVs, which are secreted under both normal and stress-induced conditions by prokaryotic and eukaryotic cells, further underscoring their critical role in parasitic survival and host manipulation (Fernandez-Becerra et al., 2023).

*Toxoplasma gondii*, the causative agent of toxoplasmosis, is a major public health problem, affecting about one-third of the human population worldwide (Dubey, 2023). Its remarkable ability to infect any nucleated cell type of all warm-blooded animals underscores its pervasive impact on both human and veterinary health (Dubey, 2008). Toxoplasmosis can have severe clinical manifestations, particularly in immunocompromised individuals and in fetal infections. Human infections predominantly arise from consuming water or food contaminated with sporulated oocysts or undercooked meat of infected livestock containing latent cysts, but also is reported to be transmitted via mother-to-child transmission. The variability in *Toxoplasma* prevalence among humans is largely attributable to socioeconomic and environmental factors, as well as cultural practices (de Barros et al., 2022; Dubey, 2023). Given its wide host range, the parasite is a major concern not only to public health, but also to the livestock industry and wildlife management as it can also compromise animal health and reproduction. It is the fourth most common foodborne parasite and a common waterborne parasite worldwide (Food and Agriculture Organization of the United Nations and World Health Organization, 2014; Almeria and Dubey, 2020). Despite its global prevalence, critical aspects of *T. gondii* pathogenesis, host-parasite interactions and immune response remain poorly understood. The lack of knowledge represents a significant barrier to the development of effective control measures and diagnostic tools.

Current research suggests that *T. gondii* exploits EVs to exert a profound influence on its host (Silva et al., 2018). The composition of *T. gondii* EVs - comprising a complex assortment of proteins, lipids and genetic material - is intricately tailored to propagate infection and ensure survival within the host (Quiarim et al., 2021). Proteomic analysis has revealed that the vesicles contain immunomodulatory molecules, which may dampen host immune defenses and promote persistent infection (Wowk et al., 2017; Ramírez-Flores et al., 2019), potentially altering host gene expression to favor the replication and dissemination of the parasite (Silva et al., 2018; Tedford et al., 2023). Despite these findings, there remain significant gaps in our understanding. A major area of uncertainty is the molecular mechanisms governing EV biogenesis, cargo sorting, and packaging. Furthermore, the functional impact of these EVs across the diverse range of host cell types that *T. gondii* can infect has yet to be characterized. To address this gap in knowledge, the EV cargo from *T. gondii* cultured in four host cell lines from different species, deliberately chosen to represent different tissue origins and species, was analyzed. This selection aimed to identify common denominators in EV composition, providing insights into conserved mechanisms of EV-mediated adaptation. The distinct environments presented by each cell type represent those encountered by *T. gondii* during systemic infections across a range of hosts. A comparison of EV composition among the cell lines will facilitate the identification of the range of adaptive strategies employed by the parasite through EVs. To gain insight into the composition of *T. gondii* EVs, we employed a multi-faceted approach, utilizing nanoparticle tracking analysis (NTA), Fourier-transform infrared spectroscopy (FTIR), transmission electron microscopy, and liquid chromatography with mass spectrometry (LC-MS/MS). This enabled us to characterize parasite EVs following replication in different host cell lines, and to conduct a quantitative analysis of the proteomic EV cargo. The findings revealed a common set of proteins in the composition of EVs isolated from tachyzoites cultivated in different environments, underscoring the significant impact of the host cell type on the composition of *T. gondii* EVs.

## 2 Materials and methods

### 2.1 Propagation of *Toxoplasma gondii* and host cell lines

*Toxoplasma gondii* RH strain parasites were grown in human foreskin fibroblasts [Hs27, obtained from ATCC CRL-1634, (Khan and Grigg, 2017)], mouse myoblasts (C2C12, ATCC CRL-1771) Vero cells [green monkey epithelial kidney cells CCL-181 (Saadatnia et al., 2010)] and intestinal porcine epithelial cells (IPEC-1, ACC 705, Leibniz Institute DSMZ-German Collection of Microorganisms and Cell Cultures GmbH, Leibniz, Germany). They were all grown in T75 flasks (VWR, Vienna, Austria) and maintained in Dulbecco’s Modified Eagle Medium (DMEM) supplemented with 10% (Hs27) and 5% fetal bovine serum (Gibco) for the other three cell lines, 100 U/ml penicillin and 0.1 mg/ml streptomycin (PAN-Biotech GmbH, Aidenbach Germany) at 37 °C in 5% CO_2._

A T25 flask was initially seeded with Hs27 cells at a density of 10,000 cells/cm². After 3 days, when the culture reached approximately 80% confluency, the cells were infected with *T. gondii* tachyzoites at a 1:3 ratio (Khan and Grigg, 2017). Tachyzoites were harvested after 2 days of infection, and 1 mL (5 × 10^6^) of the freshly egressed parasites was used to infect the target host cells. For the experiment, five T75 flasks per cell line were prepared as biological replicates. Four cell lines were seeded three days in advance at densities optimized to reach 80% confluency at the time of infection: 10,000 cells/cm² for Vero cells, 2,000 cells/cm² for myoblasts, and 25,000 cells/cm² and 10,000 cells/cm² for Hs27 cells. Following infection with *T. gondii*, the culture media was replaced 4 hours post-infection and again at 24 hours with DMEM supplemented with 3% FCS. Tachyzoites were harvested 48 hours post-infection for Hs27, Vero, and myoblast cells, and 72 hours post-infection for IPEC cells.

### 2.2 Experimental design and *T. gondii* EV isolation

For each host cell line and five biological replicate of EV isolation, we used egressed tachyzoites collected from the supernatant. Tachyzoites were centrifuged at 300 x *g* for 5 min, the pellet with the host cells was discarded. In the next centrifugation step parasites were washed twice with fresh PBS (Thermo Fisher Scientific Inc., Waltham, USA) at 600 x *g* for 10 min and at 2,000 x *g* for 10 minutes. The cleaned parasites, were counting and adjusted the number to 2.5 × 10^8^, then incubated for 2 hours in fresh (EV-free) Dulbecco’s Modified Eagle’s Medium at 37°C. After incubation the parasites were separated from their EVs with an extra step of centrifugation at 2000 x g for 10 minutes and filtering using a sterile 0.22 µm Rotilabo^®^ filter (Carl Roth, Karlsruhe, Germany). TgEVs were concentrated by several ultracentrifugation steps, first at 45,000 x *g* for 1 h at 4°C with an MLA-55 fixed-angle rotor in an Optima TLX centrifuge (Beckman Coulter, USA) to exclude potential large protein aggregates, followed by an EV pellet washing step at 55,000 x *g* for 1 h at 4°C in a TLA-45 rotor (Optima TLX centrifuge; Beckman Coulter) and finally TgEV collection at 100,000 x *g* in the TLA-45 rotor and resuspension in PBS (See **Supplementary Figure S1**) (Feix et al., 2025). The further characterization of TgEVs was performed according to MISEV guidelines (Fernandez-Becerra et al., 2023; Welsh et al., 2024).

### 2.3 Nanoparticle tracking analysis

The effective diameter and size distribution of TgEVs were measured using the ZetaView×30 TWIN Laser System 488/640 (Particle Metrix, Inning am Ammersee, Germany) as described (Comfort et al., 2021) and calibrated using 100 nm polystyrene beads. TgEVs were diluted 1:1,000 in sterile-filtered H_2_O. Particle tracking analysis was performed in scatter mode with a 488 nm laser with the following settings: Minimum brightness 30; minimum area 10; maximum brightness 255; maximum area 1000; temperature 25°C; shutter of 70 and repeated on three biological replicates with three technical replicates each.

### 2.4 Fourier-transform infrared spectroscopy

Differences in the metabolic fingerprints of TgEVs generated in different host cell environments were assessed by FT-IR spectroscopy. Therefore, purified TgEVs were subjected to FT-IR spectroscopy (Fernandez-Becerra et al., 2023). In brief, suspensions containing TgEVs were prepared and transferred to zinc selenite optical microtiter plates (Bruker Optics GmbH, Ettlingen, Germany) and dried at 40°C for 40 min. Spectra were recorded in transmission mode with the aid of an HTS-XT microplate adapter coupled to a Tensor 27 FTIR spectrometer (Bruker Optics GmbH) using the following parameters: 4000 to 500 cm−1 spectral range, 6 cm−1 spectral resolution, averaging of 32 interferograms with background subtraction for each spectrum. To compare FT-IR spectra derived from TgEVs of different cell lines, FT-IR spectra were pre-processed using vector normalization, baseline correction, and calculation of second derivates over the whole spectra using a second-order 9-point Savitzky–Golay algorithm. Spectroscopic ratios of fatty acids (3500 – 2800 cm−1), proteins (1720 – 1500 cm−1) and polysaccharides (1200 – 900 cm−1) of TgEVs of different origins were calculated as described previously (Mihály et al., 2017; Wong et al., 2022) with minor modifications. In brief, raw spectra were baseline corrected and smoothed using the Savitsky-Golay method (5 smoothing points, 3rd-grade polynomial), followed by total integration of the indicated areas. Statistical significance was calculated using an ANOVA (*p < 0.05). All experiments were repeated on five biological replicates with three technical replicates each.

### 2.5 Transmission electron microscopy

For transmission electron microscopy (TEM) imaging, pelleted parasites along with EVs were fixed in 4% neutral buffered glutaraldehyde (Merck Millipore, USA) and pre-embedded in 1.5% agar. The samples were then washed in Sorenson’s phosphate buffer (pH 6.8; Morphisto, Vienna, Austria) as described by Budik et al. (2017). Following this, they underwent post-fixation in 1% osmium tetroxide (Electron Microscopy Sciences, Hatfield Township, PA, USA). The samples were sequentially dehydrated using a graded ethanol series, soaked in propylene oxide, and embedded in epoxy resin (Serva Electrophoresis GmbH, Heidelberg, Germany). Ultrathin sections (70 nm) were prepared using a Leica Ultramicrotome (Leica Ultracut S, Vienna, Austria) and subsequently contrasted with alkaline lead citrate (Merck Millipore, USA) and methanolic uranyl acetate (Sigma Aldrich, USA). The vesicle structures were visualized using a Zeiss EM 900 transmission electron microscope (Carl Zeiss Microscopy GmbH, Jena, Germany) equipped with a digital Frame-Transfer-CCD camera (Tröndle TRS, Moorenweis, Germany).

### 2.6 Sample preparation for mass spectrometry

Radioimmunoprecipitation buffer (RIPA buffer; Thermo Fisher Scientific) was used for protein extraction. EV samples were resuspended in 50 μl of RIPA buffer and incubated at 4°C for 30 minutes on ice. Protein concentrations were then measured using the Pierce 660 nm Protein Assay (Thermo Fisher Scientific), with bovine serum albumin as the standard. Measurements were performed using a Nanodrop^®^ spectrophotometer (Thermo Fisher Scientific). 4.5 µg of the protein in RIPA buffer was reduced with 3.75 µl 200 mM tris(2-carboxyethyl) phosphine and alkylated with 3.75 µl 800 mM chloroacetaldehyde for 30 min at 37°C, respectively. SDS was added to a final concentration of 2%, and samples were then acidified with phosphoric acid to a final concentration of 1%. After adding S-Trap™ buffer (90% methanol, 100 mM tetraethylammonium bromide (TEAB), 6x the volume of sample), protein extracts were loaded onto a S-Trap™ column by centrifugation at 1000 x *g* for 1 min. Bound proteins were subsequently washed with S-Trap™ buffer at 1000 x *g* for 1 min six times to remove the SDS. After centrifugation at 4000 x *g* for 1 min to dry the column, digestion was carried out by applying 20 µl trypsin/Lys C mix (1 µg enzyme in 50 mM TEAB) onto the S-Trap™ column followed by incubation at 37°C overnight without shaking. Digested peptides were recovered with 40 µl digestion buffer (50 mM TEAB), 40 µL 0.2% formic acid and 40 µl 50% acetonitrile, respectively. The resulting peptides were then concentrated on a vacuum concentrator and taken up in 50 µl 0.1% trifluoroacetic acid (TFA). Before LC-MS analysis peptide extracts were desalted further and cleaned up using C18 spin tips (Pierce) according to the manufacturers protocol and dried in a vacuum centrifuge. The digested peptide sample was dissolved in 45 µl 0.1% TFA, 3 µl were injected to the LC-MS system, respectively.

### 2.7 Liquid chromatography with tandem mass spectrometry

Peptides were analyzed on a high-resolution Q Exactive HF Orbitrap^®^ mass spectrometer (Thermo Fisher Scientific) coupled to rapid separation liquid chromatography (nanoRSLC; Thermo Fisher Scientific) for peptide separation according to Mayr et al. (2024). Samples were injected in technical replicates.

In brief, injected peptides were trapped on a 5 mm Acclaim PepMap μ-precolumn (300 μm inner diameter, 5 μm particle size, 100 Å pore size) for sample preconcentration and desalting using 2% ACN in ultrapure water with 0.05% TFA as a mobile phase at a flow rate of 5 μL/min. Subsequent separation of peptides on a 25 cm Acclaim PepMap C18 column (75 μm inner diameter, 2 μm particle size, 100 Å pore size) was performed at a flow rate of 300 nL/min: Solvent A 0.1% FA in ultrapure water, solvent B 80% ACN with 0.08% FA. For gradient elution following parameters applied: 4% B for 0–7 min, 4–31% B from 7 to 67 min, 31–44% B from 67 to 72 min, 44–95% B from 72 to 72.1 min, 95% B until 77 min, and re-equilibration at 4% B from 78 to 90 min.

Full MS scans were acquired in positive ionization mode from 350 to 2,000 m/z at a resolution of 60,000. For subsequent fragmentation of up to 10 of the highest peaks in each MS1 spectrum, a normalized collision energy of 28 was used. Dynamic exclusion was set at 30 s. Ions with a charge of +1, +7, +8, and >+8 were excluded from fragmentation. Fragment ion spectra were detected at a resolution of 15,000. For both scan modes a maximum injection time of 50 ms applied.

### 2.8 Mass spectrometry data processing

The database search was performed using the Proteome Discoverer Software 2.4.1.15 (Thermo Fisher Scientific). The protein databases were downloaded from the UniProt homepage (http://www.uniprot.org) for the following species: *Toxoplasma gondii* RH (taxonomy ID 383379), *Homo sapiens* (taxonomy ID 9606), Mus musculus (taxonomy ID 10090), *Chlorocebus aethiops* (taxonomy ID 9534) and *Sus scrofa* (taxonomy ID 9823). Additionally, to the combined Uniprot databases, the common contaminant database cRAP was used (https://www.thegpm.org/crap/). Search settings were as follows: 10-ppm precursor mass tolerance and 0.02-Da fragment mass tolerance; dynamic modifications allowed were oxidation of methionine as well as the N-terminal protein modifications acetylation, methionine loss, and the combination of both, and static modification carbamidomethylation on cysteine. Only proteins with at least two identified peptides were reported. Intensity-based label-free quantification was applied to compare protein abundance in the experiments. Using Proteome Discoverer Software, abundance raw values were generated from mass spec raw files. Normalization to total peptide amount was performed within the software before abundance values were exported for further statistical analysis. This normalization approach sums the peptide group abundances for each sample and determines the maximum sum for all files. The normalization factor is the factor of the sum of the sample and the maximum sum in all files. Resulting normalized protein abundance values of the technical replicates were aggregated by the mean. To maintain high data quality, only proteins with abundance values for all or no biological replicate per group were considered for further data analysis performing ANOVA analysis (R Core Team, 2023). Changes in protein abundance level were considered statistically significantly up- or downregulated with a log2 fold change higher/lower than ±2 fold with a *p*-value adjusted according to Benjamini-Hochberg for controlling the false discovery rate (FDR) lower than 0.05. Only proteins identified with more than two tryptic peptides and quantified with at least one unique peptide were reported.

The mass spectrometry proteomics data have been deposited to the ProteomeXchange Consortium (http://proteomecentral.proteomexchange.org) via the PRIDE partner repository (Perez-Riverol et al., 2022) with the dataset identifier PXD055601.

### 2.9 Identification and analysis of differentially expressed proteins

TgEvs proteins with an adjusted p-value (FDR) < 0.05 in at least one of the pairwise comparisons between fibroblasts and the other cell lines, and exhibiting an absolute log2 fold change > 1, were classified as significantly differentially expressed. To further examine the expression profiles of these proteins, we applied a pattern detection algorithm using the degPatterns function from the DEGreport package (version 1.42.0; Pantano, 2024). This algorithm grouped proteins with similar expression patterns across the conditions, and the resulting clusters were sorted by size (from largest to smallest). Protein abundance values were normalized via row-wise z-transformation, in which each protein’s abundance was standardized by subtracting the mean abundance across all conditions and dividing by the corresponding standard deviation, thereby allowing for a direct comparison of expression patterns. The normalized data were visualized as an unclustered heatmap and expression pattern generated with SRplot (Tang et al., 2023).

For functional enrichment analysis of the differentially expressed proteins (DEPs), we employed ShinyGO 0.80 (Ge et al., 2020) to identify overrepresented Kyoto Encyclopedia of Genes and Genomes (KEGG) pathways and Gene Ontology (GO) terms. Enrichment p-values were calculated using the hypergeometric test, with multiple testing corrections performed via the Benjamini-Hochberg method; terms with an adjusted FDR < 0.05 were deemed statistically significant. Finally, the DEPs were ranked based on both FDR and fold enrichment to prioritize the most relevant functional categories.

## 3 Results

### 3.1 Characterization of TgEVs from tachyzoites derived from different host cells

**Figure 1:**
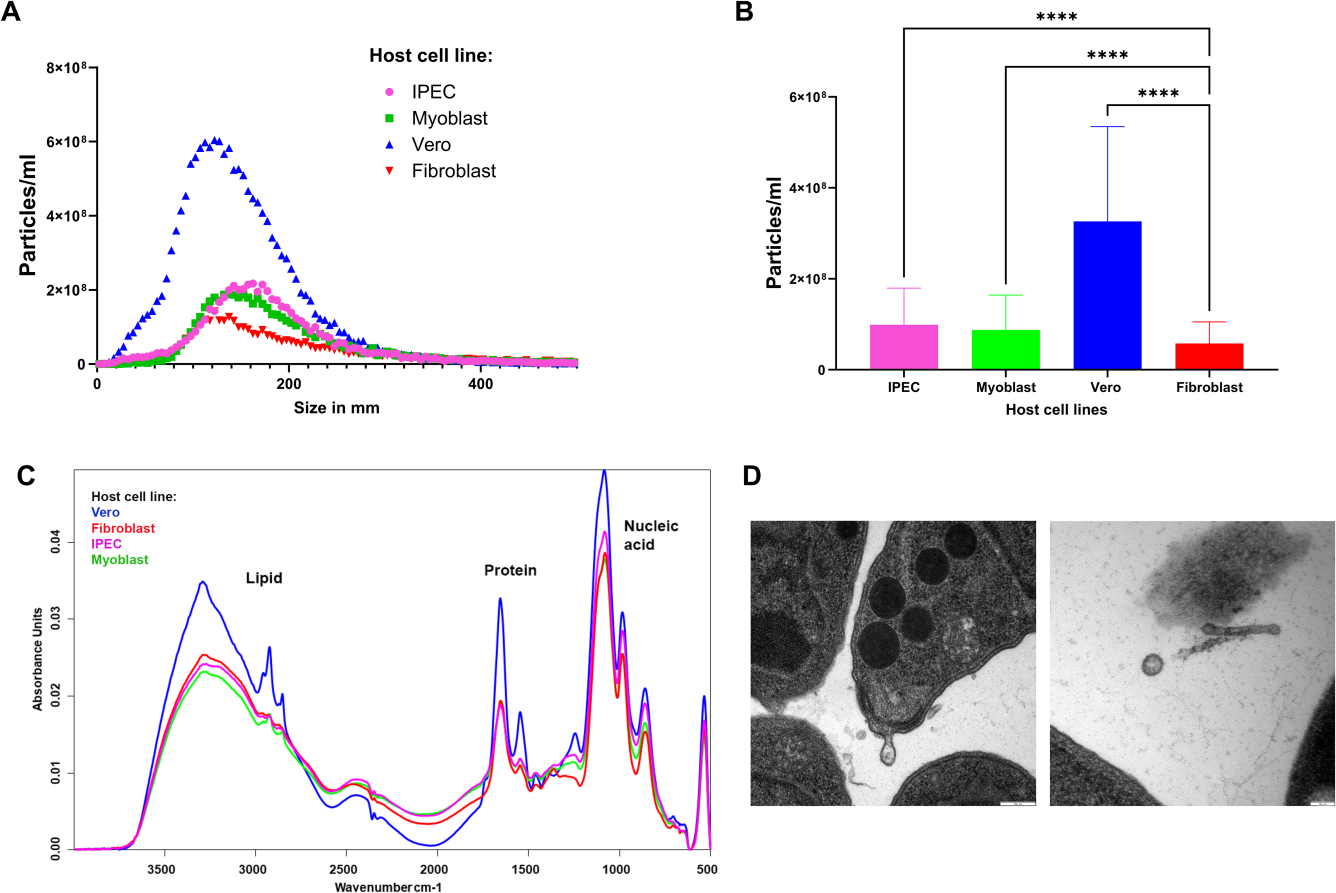
Isolation and characterization of EVs of *T. gondii.* **(A)** Distribution of EV size (nm) and particles/ml measured by NTA. The data represent the three reads of five biological replicates. **(B)** Concentration (particles/ml), values represent the mean ± standard deviation (SD). Asterisks represent significant difference (****P<0.0001). **(C)** The insets show FT-IR spectra for lipid (2800-3000 cm^-1^ for C-H stretching) and protein regions (1500-1700 cm^-1^, represented as amide I and II bands). **(D)** Transmission electron microscopy images of EVs and tachyzoites of *T. gondii* grown in the fibroblast cell line. Scale bar: 100nm.

In an initial pilot study, a protocol for isolating EVs from *T. gondii* (*Tg*EVs) was refined to yield sufficient EV material for quantification, separation, and proteomic analysis. To evaluate the efficacy of our isolation protocol, we employed NTA, FT-IR and TEM. NTA analysis revealed that identical amounts of parasites shed varying numbers of particles of different sizes on the range of the exosomes, depending on the host cell line (**Figures 1A, B**). Specifically, *Tg*EVs cultivated in Vero cells exhibited a higher concentration and a broader size distribution compared to those from IPEC, myoblast, and fibroblast cells. *Tg*EVs from Vero and fibroblast cells displayed a more heterogeneous population, including smaller vesicles, whereas those from IPEC and myoblast cells appeared more uniform and larger in size.

FT-IR spectroscopy was employed to assess the molecular composition of the isolated *Tg*EVs. The spectra indicated distinct lipid, protein, and nucleic acid profiles for *TgEVs* from each host cell line (**Figure 1C**). FT-IR analysis revealed characteristic absorbance peaks corresponding to lipids, proteins, and nucleic acids. For example, the lipid region (2800-3000 cm^-1) and protein region (1500-1700 cm^-1), showed notable differences with Vero cell-derived *Tg*EVs displaying higher absorbance. In particular, the lipid region showed two distinct patterns among the *Tg*EVs derived from different host cells. EVs from fibroblast and IPEC cells exhibited similar lipid absorbance profiles, indicating comparable lipid content. In contrast, *Tg*EVs from myoblast and Vero cells displayed a different lipid absorbance pattern, suggesting higher lipid content in these *Tg*EVs. Additionally, the nucleic acid region (900-1200 cm^-1) had pronounced peaks in *Tg*EVs from Vero cells, suggesting variations in nucleic acid content. These spectral differences indicate the influence of the host cell environment on the biochemical composition of *TgEVs*. Transmission electron microscopy (TEM) offered detailed morphological characterization of the TgEVs. The images confirmed the presence of typical vesicular structures and revealed variations in size and shape among EVs from different host cell lines (**Figure 1D**; **Supplementary Figure S2**). TEM also provided clear visualization of the intact bilayer membranes of the EVs and suggested that they are preferentially shed at the apical end of tachyzoites (personal observation).

### 3.2 EV proteomes of *T. gondii*

**Figure 2:**
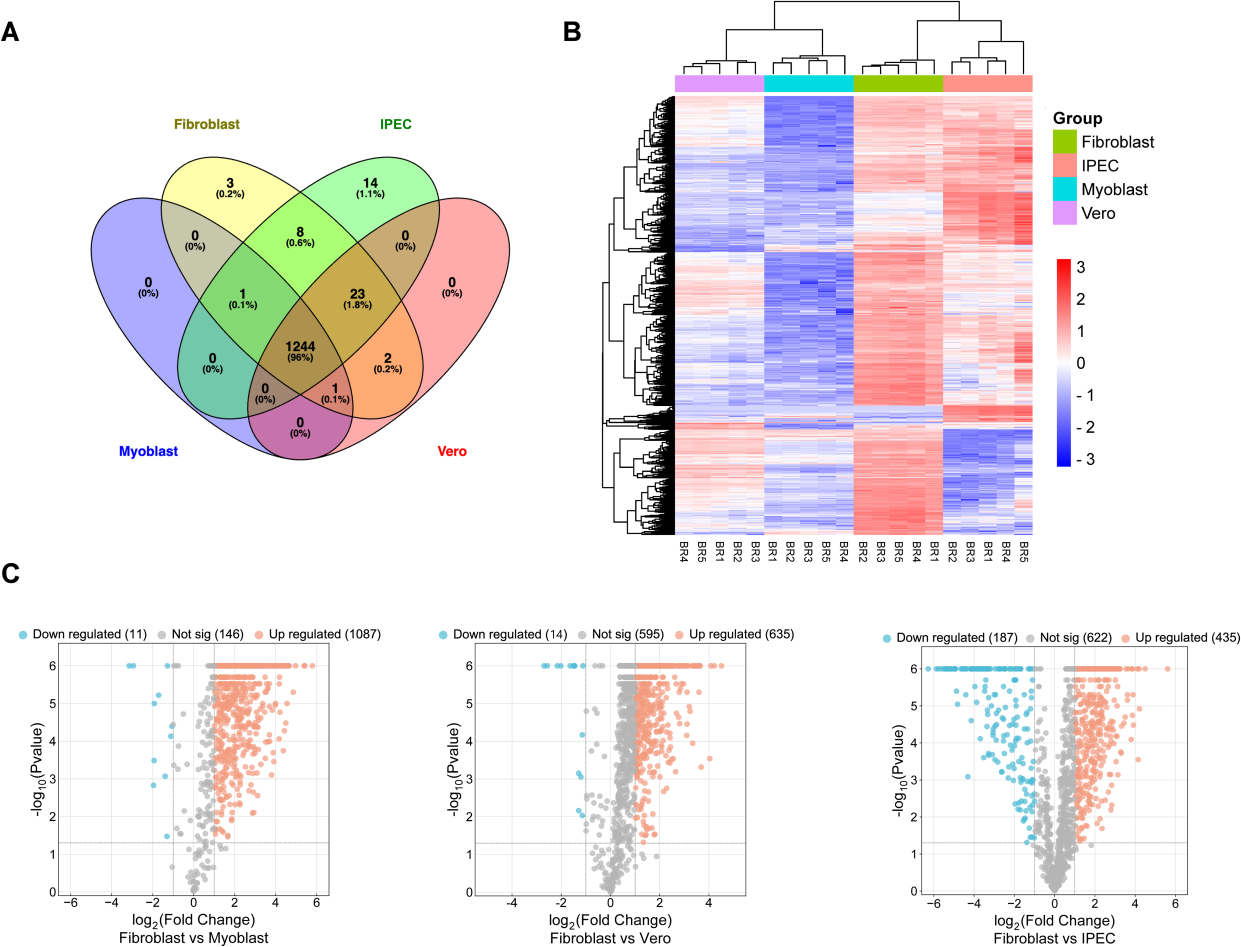
Proteomic analysis of EVs released from tachyzoite stages of *T. gondii* infection. **(A)** Venn Diagram illustrates the overlap of protein markers between the EVs isolated from four different host cell lines: fibroblasts, myoblasts, intestinal porcine epithelial cells (IPEC), and Vero cells. The numbers within the diagram sections indicate the count and percentage of unique and shared proteins among these cell lines. **(B)** Clustered heatmap showing row-wise z-transformed protein levels across four groups. Each row represents a distinct protein identified within the EVs. The colour scale indicates expression levels, with red representing higher than average expression, white indicating average expression, and blue signifying lower than average expression. The x-axis lists the different host cell lines (myoblasts, fibroblasts, IPEC, and Vero) with the mean values of five biological replicates for each cell line, labelled R1–R5. **(C)** Volcano plots show the differential expression of proteins in T. gondii EVs when comparing fibroblasts with each of the other host cell lines: Myoblast, Vero and IPEC. Each dot represents a protein, with the x-axis showing the log2 fold change in expression and the y-axis showing the -log10 p-value of the change in expression. Significantly upregulated proteins are shown in red, significantly downregulated proteins are shown in blue and non-significant proteins are shown in grey. The numbers in brackets indicate the total number of proteins in each category for each comparison.

To understand the proteomic changes in the *Tg*EVs, the protein composition was analyzed using LC-MS/MS analysis to quantify and compare protein abundance. We optimized the sTRAP method for protein digestion prior to peptide separation. In total, 2,664 proteins were identified with at least two peptides, of which at least one peptide was unique per protein. After filtering out contaminant proteins from the cell lines, the samples yielded a combined dataset of 1,833 proteins assigned to the *T. gondii* genome. Of these, 1,392 proteins were reliably quantified, meaning that they were detected in all five biological replicates of at least one experimental group, allowing for robust comparative analyses. These proteins were then subjected to hypothesis testing. A total of 1,296 proteins were identified as significantly differentially expressed in at least one of the four tested contrasts, using a global FDR cut-off of 5% and a minimum absolute log2 fold change of one (**Table 1**). The Venn diagram (**Figure 2A**) illustrates the overlap of DEPs among the four host cell lines. Principal component analysis on these 1,296 proteins showed clustering according to their host cell along the first and second axes, explaining 44.2% and 33.0% of the variance, respectively (**Supplementary Table 1**). There were 1,244 proteins (95.98%) common across all host cell lines, indicating a substantial core *Tg*EVs protein cargo shared by *T. gondii*-infected cells. Unique protein profiles were also identified, with fibroblasts, IPEC, and Vero cells exhibiting exclusive proteins (3, 14, and 2 proteins, respectively), while no unique proteins were detected in the myoblasts (**Figure 2A**; **Supplementary Table 1**). These 1,244 proteins were sorted according to their abundance pattern-cluster membership (**Figure 2B**). Notably, *Tg*EVs proteins from IPEC and fibroblast cells clustered closely together, suggesting a more similar protein expression profile compared to myoblasts and Vero cells. To further investigate the differential expression of *Tg*EVs proteins, we generated Volcano plots for each pairwise comparison between fibroblasts and the other host cell lines (**Figure 2C**). Volcano plot analyses showed significant upregulation of proteins in fibroblast derived *Tg*EVs: 1,087 proteins were upregulated and 11 downregulated compared to myoblasts; 635 proteins upregulated and 14 downregulated compared to Vero cells; and 435 proteins upregulated and 187 downregulated compared to IPEC cells (**Supplementary Table 2**).

**Table 1:** Summary table of the differential expression analysis of 1,244 proteins of *T. gondii* EVs.

1,244 proteins > significant at 5% FDR and a minimum effect size of abs(log2(FC))>1			
Contrast	Fibroblast vs IPEC	Fibroblast vs Vero	Fibroblast vs Myoblast
**Not significant**	622	595	146
**Upregulated**	435	635	1087
**Downregulated**	187	14	11

We compared fibroblast versus IPEC, Vero and myoblast host cell lines. We show the number of significantly up- or downregulated contrast at 5% FDR with a minimum effect size of absolute log2 fold change >1 and the number of not differentially expressed proteins.

### 3.3 Protein composition, EVs markers and GO terms

**Figure 3:**
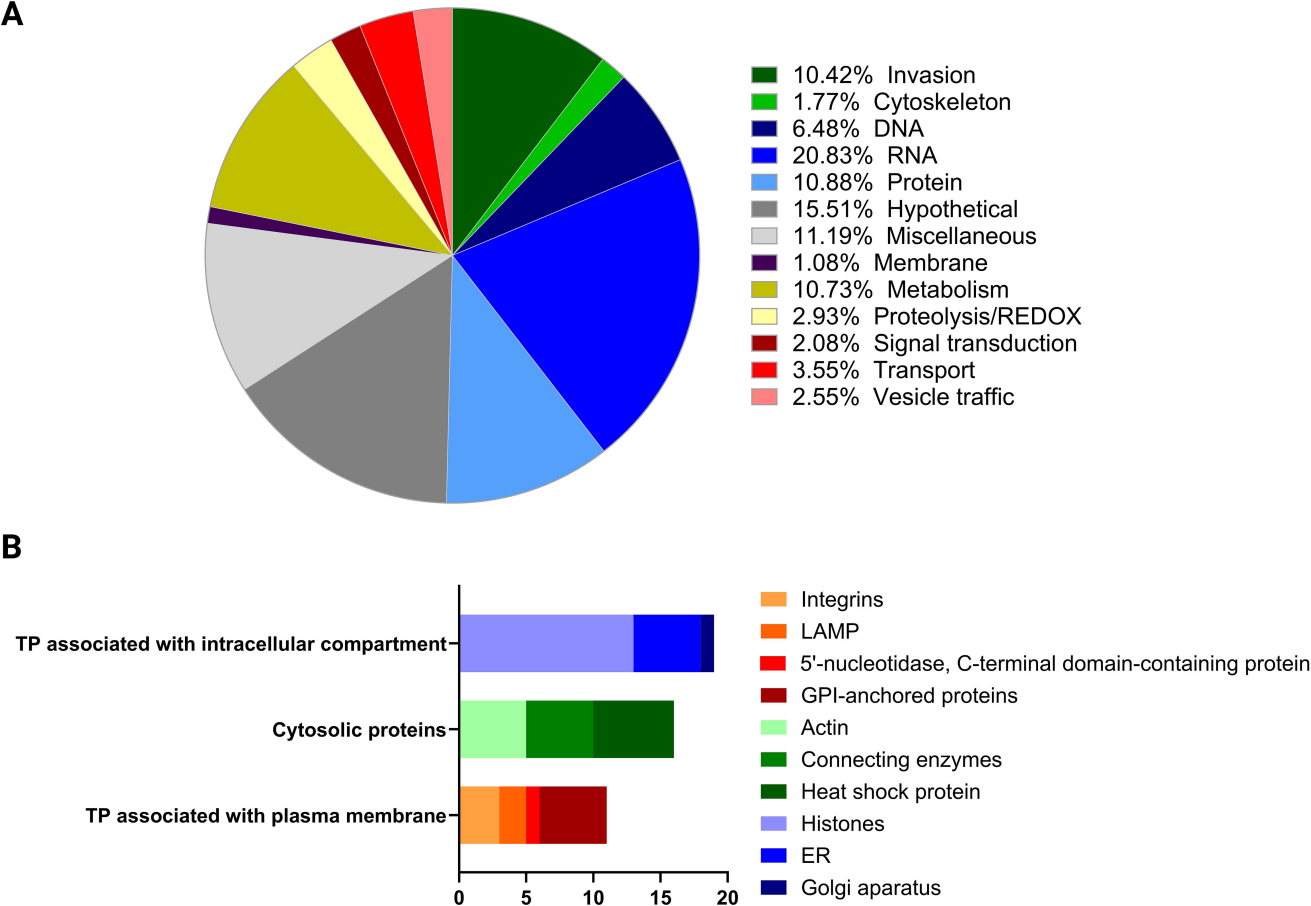
Characterization of EV protein content based on protein distribution. **(A)** The pie chart depicts the biological functions categorization of proteins found in EVs. The biological function of proteins coded by each of the 1,244 proteins was assigned manually based on ToxoDB, Blast2Go, KEGG or annotations published previously highlighting the relative abundance of different functional groups. **(B)** The bar chart illustrates the distribution of EV proteins marker categorized by their association with specific cellular components. The number of proteins in each category is indicated along the x-axis.

The 1,244 proteins identified were classified into functional categories, including 201 hypothetical and 137 miscellaneous proteins. Among the proteins related to invasion, 136 were identified, with 92 being secreted proteins. These included micronemal proteins (MIC), rhoptry-related proteins (ROP and RON), dense granule proteins (GRA), and 29 SAG-related proteins. Additionally, 26 cytoskeleton proteins such as actin, myosin, tubulin, and clathrin were detected, along with 33 vesicle traffic-related proteins including Ras and Rab proteins. The category associated with RNA processes was well-represented with 270 proteins, alongside proteins involved in DNA and protein-related processes with a total of 224 proteins. Furthermore, 27 proteins were identified as being involved in cell-signaling processes, and 139 proteins were associated with metabolism (**Figure 3A**; **Supplementary Table 1**). Previously characterized EV protein markers were detected. We reported 75 proteins including Rab and Ras related proteins, SNARE, heat shock proteins, actin and tubulin, GAPDH, histones and proteins related to the Golgi apparatus, endoplasmic reticulum, mitochondria and membrane proteins (**Figure 3B**).

**Figure 4:**
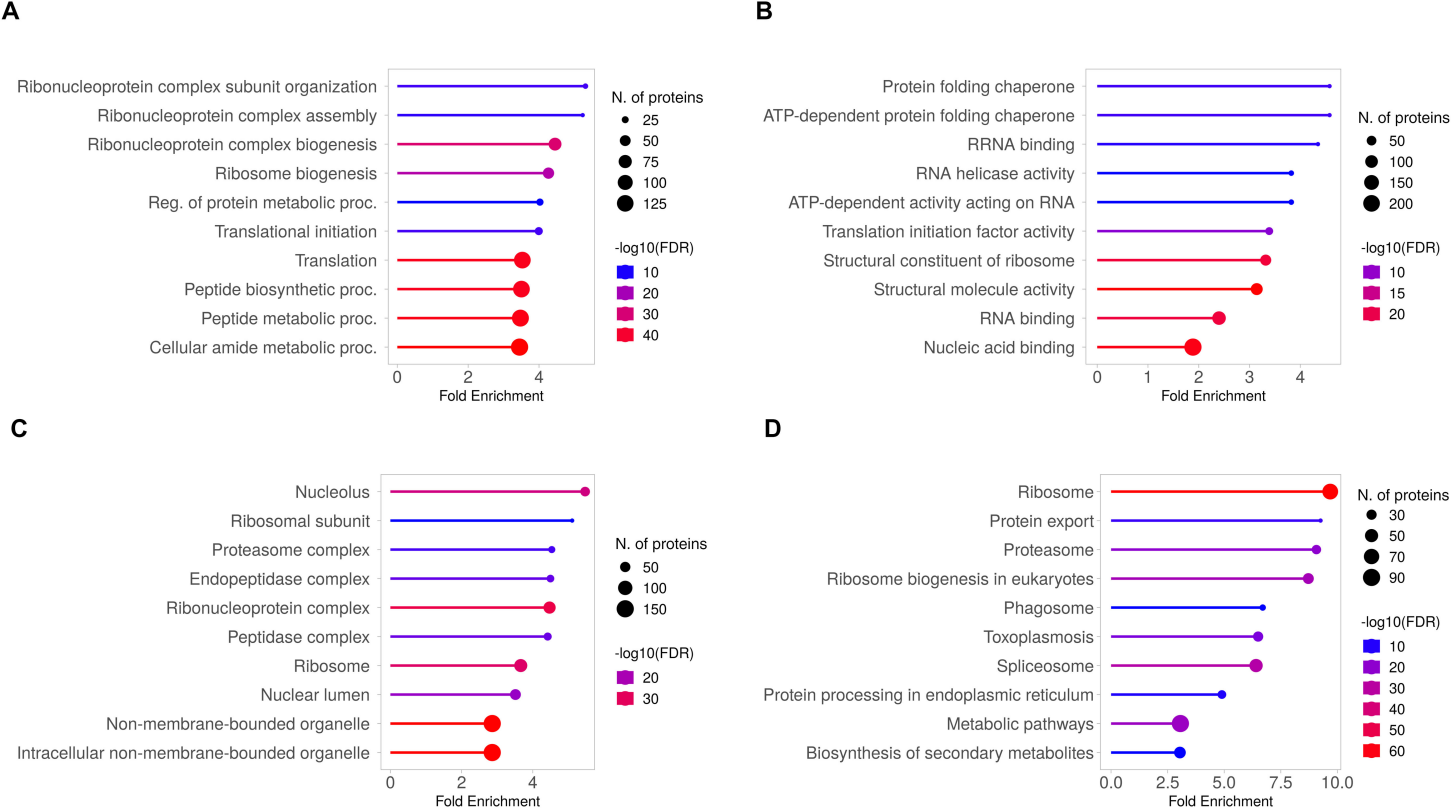
GO and KEGG pathway analysis of the 1,244 shared proteins identified in *T. gondii* EVs. The top 10 enriched GO terms are presented for the differentially expressed proteins, categorized into three main groups: **(A)** Biological Process, **(B)** Molecular Function and **(C)** Cellular Component. Each GO category shows the identified functions, with corresponding numbers of differentially expressed proteins and fold enrichment values. **(D)** KEGG pathway analysis, illustrating the top enriched pathways. The X-axis represents the fold enrichment of the pathway, and the Y-axis lists the major pathways enriched in the dataset. Circle size reflects the number of proteins involved in each pathway, and the colour gradient represents -log10(FDR).

To investigate the functional implications, we performed GO term enrichment analysis on the common set of *Tg*EVs proteins (**Figure 4**). For each GO category the ten significant terms with the lowest p-values are displayed. Biological Process GO term enrichment analysis revealed significant enrichment of proteins associated with ribonucleoprotein complex subunit organization, ribonucleoprotein complex assembly, and ribosome biogenesis (**Figure 4A**). Other significantly enriched processes included regulation of protein metabolic processes, translational initiation, translation, peptide biosynthetic process, peptide metabolic process, and cellular amide metabolic process. Molecular Function GO analysis revealed significant enrichment in protein folding chaperone activity, ATP-dependent protein folding chaperone activity, and RNA helicase activity (**Figure 4B**). Additional enriched functions included ATP-dependent activity acting on RNA, translation initiation factor activity, structural constituent of ribosome, and nucleic acid binding. For cellular components, the enriched terms included nucleolus, ribosomal subunit, proteasome complex, endopeptidase complex, and ribonucleoprotein complex (**Figure 4C**). Other significant components were peptidase complex, ribosome, nuclear lumen, non-membrane-bounded organelle, and intracellular non-membrane-bounded organelle. These GO terms showed fold enrichments between 2 and 4, with the number of genes involved ranging from 50 to over 150. KEGG pathway analysis revealed significant enrichment in pathways such as ribosome, protein export, proteasome, ribosome biogenesis in eukaryotes, and phagosome (**Figure 4D**). Other enriched pathways included toxoplasmosis, spliceosome, protein processing in endoplasmic reticulum, metabolic pathways, and biosynthesis of secondary metabolites. The fold enrichment for these pathways varied from 2.5 to 7.5, with the number of genes ranging from 30 to over 90.

### 3.4 Quantitative comparison of EV-derived proteins

The proteomic analysis revealed distinct patterns of protein abundance when comparing fibroblast-derived *TgEVs* with those from other host environments. In the fibroblast *vs*. myoblast *Tg*EVs comparison, we identified only 14 proteins with higher abundance in myoblast-derived *Tg*EVs, primarily related to DNA and RNA processing. In fibroblast- *Tg*EVs, we observed a significant enrichment of proteins involved in cytoskeleton, invasion mechanisms, metabolism, protein processing, DNA and RNA processing, and vesicle trafficking compared to those from the Vero cell environment. In contrast, DNA-related proteins, particularly histones, were found in higher abundance in the Vero cell environment. Overall, fibroblast-derived *TgEVs* showed an upregulation of proteins associated with invasion, metabolic pathways, ribosomal proteins, RNA processing, and vesicle trafficking. Conversely, *Tg*EVs derived from IPEC cells exhibited an enrichment of proteins involved in DNA processing, RNA processing, translation, and protein phosphorylation (**Supplementary Table 2**).

**Figure 5:**
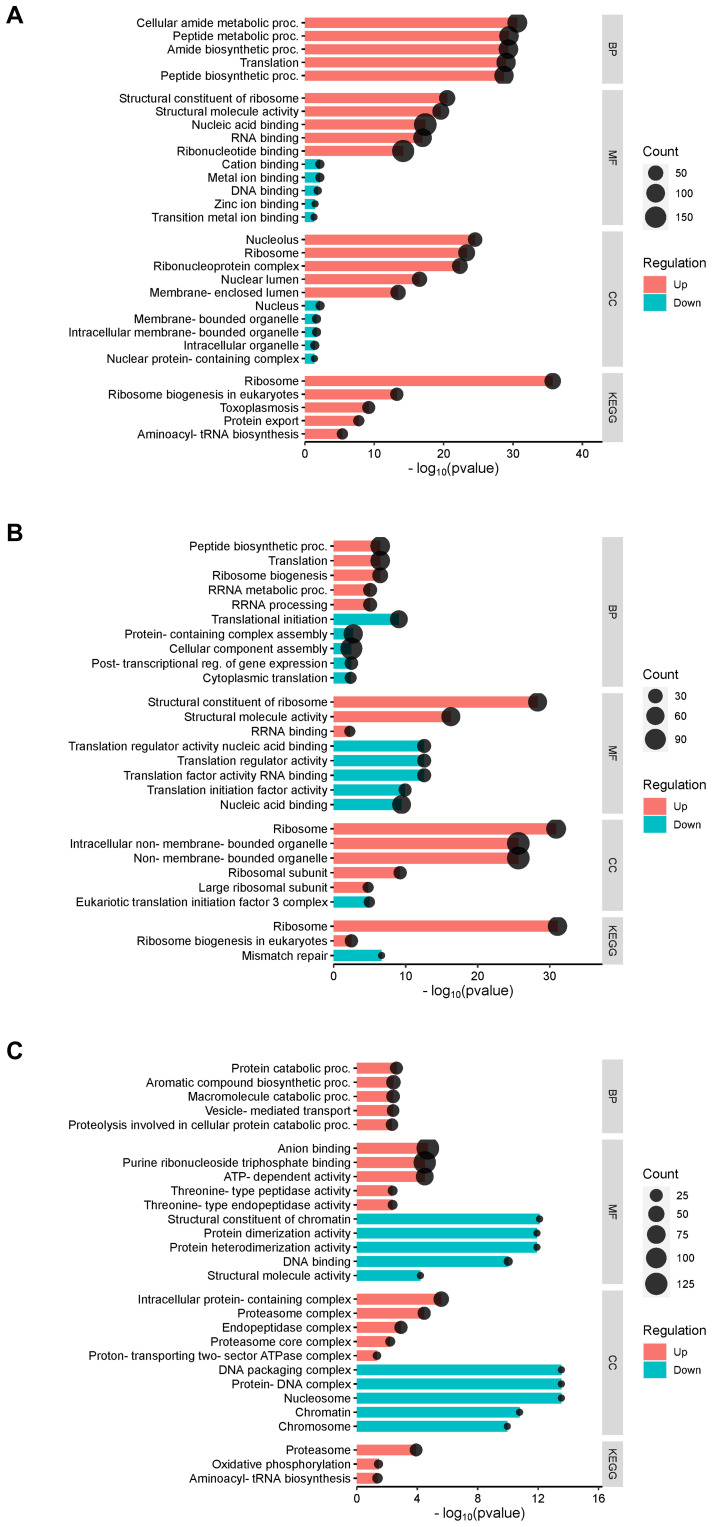
GO Term enrichment analysis of differentially expressed proteins in *T. gondii* EVs across different host cell lines. **(A)** Fibroblast vs myoblast. **(B)** Fibroblast vs IPEC and **(C)** Fibroblast vs Vero. In all panels, the x-axis represents the -log10(p-value) of the enrichment, indicating the statistical significance, while the size of the dots represents the count of proteins associated with each term. Red bars indicate upregulated proteins, and blue bars indicate downregulated proteins. The analysis uses the total set of common EV proteins as the background for enrichment calculations.

To elucidate the functional implications, we performed GO term enrichment analysis on the upregulated and downregulated proteins from each comparison, using the total set of common *Tg*EVs proteins as the background. In the comparison between fibroblast and myoblast host cells (**Figure 5A**), our analysis revealed significant enrichment of membrane-associated proteins among the upregulated proteins, while downregulated proteins were enriched in DNA repair mechanisms. For the comparison between fibroblast and IPEC host cells (**Figure 5B**), upregulated proteins showed enrichment in processes related to protein synthesis and ribosomal assembly, whereas downregulated proteins were linked to DNA repair mechanisms. Upregulated proteins were also associated with ribosomal and other organelle components, while downregulated proteins showed decreased association with translation and nucleic acid binding functions. In the fibroblast versus Vero cell comparison (**Figure 5C**), upregulated proteins were enriched in protein degradation and transport mechanisms. Downregulated proteins were associated with DNA packaging and chromatin organization. Upregulated proteins also showed enrichment in components involved in protein degradation, while molecular functions included increased binding and catalytic activities. Downregulated proteins were linked to chromatin structure and DNA binding activities.

### 3.5 Expression patterns of invasion related proteins in *T. gondii* EVs

**Figure 6:**
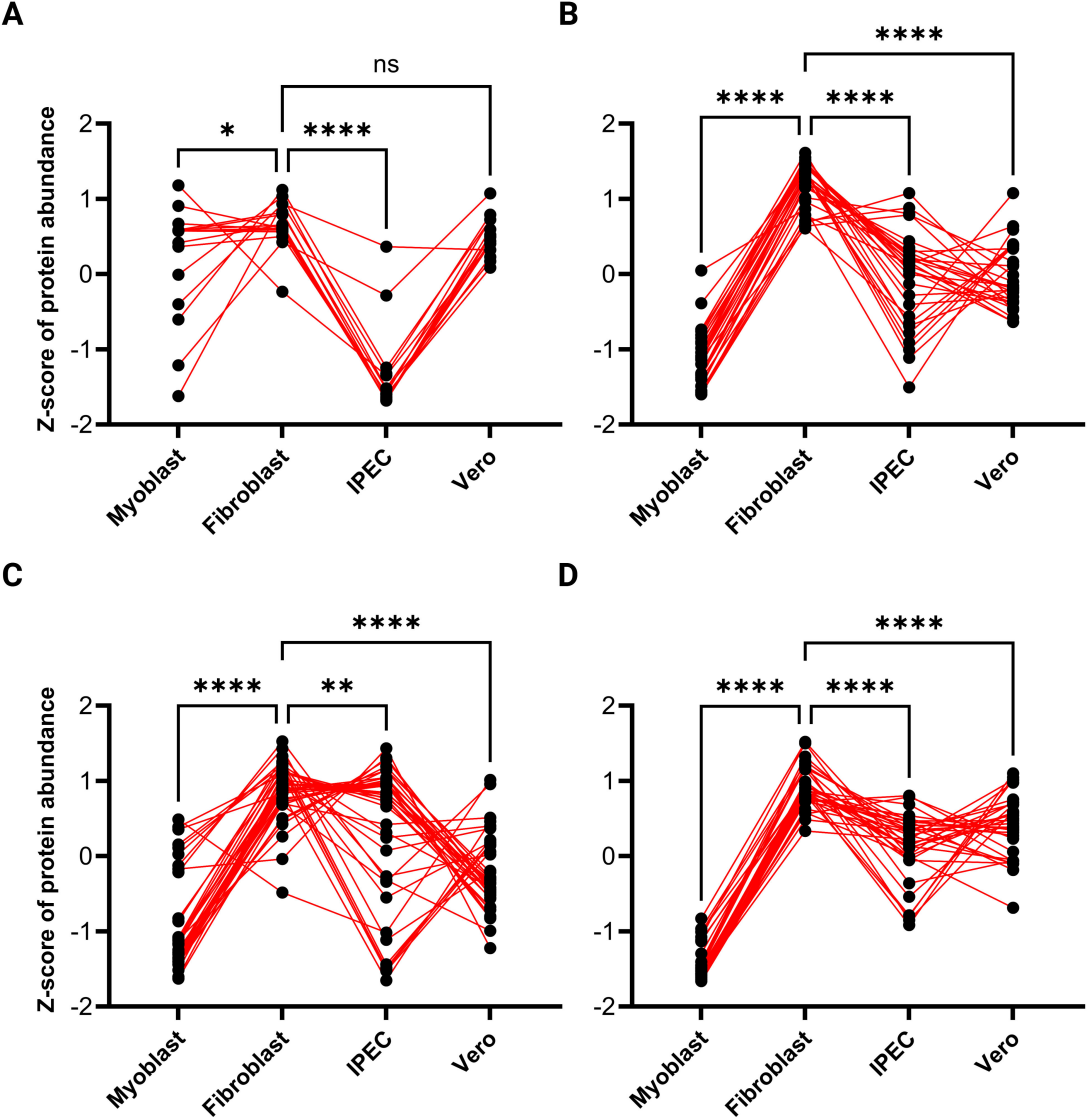
Expression patterns of invasion-related proteins in *T. gondii* EVs across different host cell lines. This figure illustrates the relative expression patterns of invasion-related protein groups in *T. gondii* EVs derived from four distinct host cell lines (myoblasts, fibroblasts, IPEC cells, and Vero cells). The panels represent: **(A)** microneme proteins, **(B)** SAG (surface antigen) proteins, **(C)** rhoptry proteins, and **(D)** dense granule proteins. The Z-scores of protein abundance were calculated to normalize and compare the relative expression levels across cell types. Each dot represents a single protein, and red lines connect the same protein across cell types, visualizing variations in abundance between host environments. Statistical significance for pairwise comparisons is indicated as follows: *P < 0.05, **P < 0.01***, P < 0.001, ****P < 0.0001; ns denotes no significant difference.

To investigate the expression patterns of invasion-related proteins in *TgEVs*, a four-group analysis was conducted, comprising microneme proteins (MIC), SAG (surface antigen) proteins, rhoptry proteins (ROP and RON), and dense granule proteins (GRA) (see **Supplementary Table 3**). These proteins were subjected to a pattern detection algorithm with detected patterns shown in **Figure 6** and then sorted according to their pattern-cluster membership. MIC proteins showed variations in abundance between host cell lines. A significant decrease in expression levels was observed in *TgEVs* from IPEC samples. In contrast, *TgEVs* from fibroblasts and Vero cells showed significantly higher expression levels, with no notable differences between these two groups, suggesting an enrichment of microneme proteins in these cell types. *T. gondii* EVs from myoblast samples showed intermediate expression levels, with a slight downregulation compared to fibroblast and Vero samples (**Figure 6A**). SAG proteins showed variability in expression between cell lines. *T. gondii* EVs derived from IPEC, Vero and myoblast samples showed lower expression levels of SAG proteins, whereas fibroblast-derived *TgEVs* showed significant upregulation (**Figure 6B**). Rhoptry proteins showed a distinct pattern of expression, with significant upregulation in *TgEVs* from fibroblasts and IPEC samples, followed by downregulation in EVs from myoblasts and Vero samples (**Figure 6C**). GRA proteins also showed variability between the different host cell lines. *TgEVs* from myoblast samples showed significantly reduced expression compared to the other cell lines. In contrast, *TgEVs* from fibroblasts showed the highest expression levels, while *Tg*EVs from IPEC and Vero cells showed relatively stable expression with a slight upregulation compared to myoblast samples (**Figure 6D**).

## 4 Discussion

There is growing evidence that the host cell environment plays a critical role in *T. gondii* biology. In this study, we demonstrated the adaptability of *T. gondii* to its cellular environment by showing that the protein cargo of its EVs shifts after growth and egress in different host cell types, including those commonly used in *T. gondii* research and a porcine intestinal epithelial cell.

Understanding the role of EVs in parasite biology is an emerging frontier in parasitology. Once regarded as cellular debris, EVs are now recognized as critical mediators of intercellular communication, capable of influencing a range of biological processes (Sibley et al., 1986; Blader and Saeij, 2009). The observed flexibility in EV protein composition may be a key factor behind the ability of *T. gondii* to infect nucleated cells across a broad range of warm-blooded hosts with varying susceptibilities to clinical toxoplasmosis. This adaptability allows *T. gondii* to thrive in diverse environments, infecting highly susceptible hosts like primates as well as more resistant species such as pigs (Dubey, 2023; Dubey et al., 2020).

### 4.1 Influence of host cell line on EV size and composition

Initial analyses were carried out to characterize the TgEVs derived from tachyzoites cultured in four different host cell lines. Using NTA, FT-IR and TEM, we observed consistent EV release behavior across all host cell lines. NTA revealed that although identical amounts of *T. gondii* were used, the number and size of TgEVs varied significantly between host cell lines. Herein it is shown two classes of vesicles with range sizes of 50-200 nm, similar to those found in previous studies (Silva et al., 2018; Quiarim et al., 2021), which were classified according to their sizes as small EVs according to MISEV 2023 (Welsh et al., 2024). In particular, the particle size distribution and number of TgEVs differed significantly between the different host cell lines, suggesting that *T. gondii* may adjust its EV production as an adaptive mechanism to optimize host-parasite interactions. FT-IR analysis further supported these observations by revealing distinct EV spectral fingerprints for each host cell line, demonstrating differences in the lipid and protein composition of the TgEVs. These differences, as shown by the unique FT-IR spectral profiles suggest that TgEVs derived from different host cells have different biochemical compositions. Such variations in lipid and protein content could influence the biological functions of EVs, including membrane fusion, signaling and/or immune modulation, thereby affecting host-pathogen interactions. TEM images combined with NTA data revealed that EVs produced by *T. gondii* ranged in size from 100 to 200 nm, resembling microvesicles. These TEM results are consistent with previous studies on *T. gondii* EVs (Ramírez-Flores et al., 2019; Quiarim et al., 2021; Silva et al., 2018), which reported that tachyzoites spontaneously release numerous EVs into the extracellular environment, particularly from the apical and posterior regions, as well as from the plasma membrane.

### 4.2 Proteomic analysis and differential expression

The proteomic analysis of *Toxoplasma gondii* EVs revealed a conserved core of 1,244 proteins consistently identified across all host cell lines studied. This significantly exceeds the number of proteins reported in previous studies, which identified 321 proteins of ectosomes and exosomes (Ramírez-Flores et al., 2019) and 340 proteins (Wowk et al., 2017). Furthermore, except for a small fraction (~2.5%, **Supplementary Figure S3**), almost all proteins identified in these previous studies are also detected in our core proteomic data, highlighting both the coverage and the reproducibility of those results. The higher protein yield in our study likely reflects the use of the optimized S-Trap method (HaileMariam et al., 2018), which enhances protein digestion efficiency compared to the traditional SDS-gel band digestion approach used in earlier analyses (Silva et al., 2018; Wowk et al., 2017; Quiarim et al., 2021). The broad functional diversity of EV proteins underscores the complex role of TgEVs in modulating host cell biology (Vicentini et al., 2024). The significant proportion of proteins involved in RNA and DNA processes, invasion, and protein metabolism suggests that TgEVs are crucial vehicles for delivering key molecular signals that facilitate parasite survival, replication, and immune evasion. Our findings align with previous studies, which also identified a similar pattern of protein categories (Quiarim et al., 2021; Ramírez-Flores et al., 2019). Notably, proteins associated with ribosomes constitute the largest category, as previously described in *T. gondii* ectosomes and exosomes (Ramírez-Flores et al., 2019) further validating the consistency and importance of these components in *T. gondii* EVs. In addition, these TgEVs deliver virulence factors to the host, including key invasion proteins such as SAG, MIC, GRA, and ROP/RON, as demonstrated in previous studies (Ramírez-Flores et al., 2019; Torrecilhas et al., 2012; Vicentini et al., 2024; Quiarim et al., 2021).

The core set of proteins were also analyzed to characterize the EVs biomarker, following the five-component framework introduced in MISEV2023 (Welsh et al., 2024). Our results reveal the presence of proteins associated with intracellular compartments, the cytosol, and the plasma membrane associated proteins. We detected in our samples actin and tubulin, Rab and Ras proteins as previously described in *T. gondii* EVs (Ramírez-Flores et al., 2019). In addition to these, other proteins such as heat-shock proteins (HSPs), thioredoxin peroxidase, metalloprotease, and glutathione S-transferase have also been identified in parasite-derived EVs. In our results we found Clathrin, SNARE, histones, enolase 2, and GAPDH, which have not been previously described in *T. gondii*, are known EV biomarkers in mammalian cells (Welsh et al., 2024; Kim et al., 2015, 2013; Kalra et al., 2012). The enrichment of nucleus-associated proteins suggests that *T. gondii* EVs may play a role in altering host gene expression (Zhou et al., 2024), while the presence of cytoskeletal and membrane proteins could facilitate the remodeling of host cell architecture to favor parasite invasion and intracellular survival (Sung et al., 2021). The results of the KEGG and GO- biological process (BP) analysis indicated a notable increase in the representation of categories associated with ribonucleoprotein complex subunit organization, ribosome biogenesis, and translational initiation. In addition, the molecular function (MF) analysis revealed a significant enrichment of proteins involved in RNA binding, RNA helicase activity, ATP-dependent activity acting on RNA, structural constituent of ribosome and protein folding chaperones and the cellular component are enriched in proteins involved ribosome and ribosomal subunit. This indicates that *T. gondii* EVs may be a crucial factor in regulating host cell processes, particularly by stabilizing RNA and enhancing translation efficiency. Furthermore, it suggests that TgEVs play a pivotal role in modulating the regulation of ribosome assembly and function, which in turn reprograms receptor cells and alters their phenotype (Ochkasova et al., 2023). In addition, the presence of proteasome-related activity and peptidase complexes indicates the potential involvement of TgEVs in protein degradation pathways. This suggests that *T. gondii* may utilize these vesicles to regulate protein turnover and maintain cellular homeostasis (Ben-Nissan et al., 2022) or a similar role as in *P. falciparum* in which the 20S proteome of the EVs altered the membrane stiffness of red blood cell to facilitate malaria parasite growth (Dekel et al., 2021).

### 4.3 Host-cell environment

*Toxoplasma gondii* encounters a wide variety of cellular environments when infecting different host cell types. Each of these cell types provides the parasite with distinct ecological niches, influenced by their unique physiological and metabolic characteristics (Swierzy et al., 2017). These variations have a significant impact on the protein composition of the EVs released by *T. gondii*, reflecting the ability of the parasite to adapt to and exploit its host. Although *T. gondii* strains have different growth rates in various cell lines (Evans et al., 1999), human foreskin fibroblast cells have been utilized widely as the primary cell line to maintain *in vitro* cultures of *T. gondii* (Khan and Grigg, 2017). Fibroblasts are the most common cells of connective tissue in animals (Plikus et al., 2021). Besides their commonly known role as structural components, fibroblasts play a critical role in an immune response, functioning as sentinel cells that respond to infections or tissue damage by producing cytokines, such as IL-6 and IL-8, and chemokines, like CXCL10. They can be activated by IFN-γ, triggering anti-parasitic mechanisms, such as tryptophan degradation, which can inhibit the growth of intracellular pathogens (Cavagnero and Gallo, 2022). Fibroblasts, as metabolically active cells, offer a permissive environment for *T. gondii* replication, making them a valuable model in host-pathogen interaction research due to their ease of culture and robust characteristics (Mital et al., 2005). With a doubling time of 24 hours, fibroblasts create a nutrient-rich and favorable environment that facilitates efficient invasion, replication, and survival. In this setting, *T. gondii* likely optimizes its EVs composition to invade surrounding cells, as suggested by the high abundance of invasion related proteins compared to the other host-cell environments, indicating also a potential strategy for more effective manipulation of host cell functions. A strong enrichment of processes related to protein biosynthesis, including peptide biosynthetic processes, ribosome biogenesis, and translation, reflects a robust support for TgEVs enriched in invasion-related and translational machinery proteins. The second host cell line, Vero cells, are widely used in infectious disease research because of their permissiveness to pathogens. They lack certain antiviral responses, such as interferon production, making them susceptible to a variety of intracellular pathogens. This weak immune activation makes Vero cells particularly useful for studying *T. gondii* (Evans et al., 1999). Myoblasts, skeletal muscle precursor cells form the third preferred tissues for persistence of *T. gondii* and experimental evidence indicate that myoblast are an appropriate cell type for *T. gondii* stage conversion and tissue cyst formation (Swierzy et al., 2014), primarily function in repair and regeneration rather than immune defense. Although they can produce some inflammatory mediators like IL-6, they are generally less immunologically active compared to fibroblasts or IPEC. In these two faster-propagating cells, myoblasts (Yaffe and Saxel, 1977) and Vero cells (Ammerman et al., 2008), which have an average doubling time of 17 and 22 hours respectively, and are metabolically more active than fibroblast, *T. gondii* releases EVs enriched in proteins that support rapid parasite replication and cellular proliferation. The increased abundance of proteins involved in cell cycle progression, chromatin assembly (histones), chromosome organization and RNA biosynthesis proteins in *TgEVs* from Vero cells likely are indicative for active replication compared to fibroblast. Histones could play a role in modulating gene expression, immune responses, or cellular stress pathways, creating more optimal conditions for parasite survival and replication (Zhou et al., 2024). In contrast, myoblasts, might present a less restrictive environment compared to fibroblast for *T. gondii*. This reduced immunological activity may limit the necessity for *T. gondii* to secrete EVs rich in proteins for immune modulation or invasion. Furthermore, while myoblasts are metabolically active, their primary function is not to provide the permissive environment seen in fibroblasts or the nutrient-rich conditions characteristic of Vero cells. Instead, the environment in myoblasts may present fewer resources for the parasite, leading to a lower release of *Tg*EVs or a downregulation of EV cargo synthesis. This is consistent with the observed reductions in invasion-related proteins, such as micronemes, dense granules, and rhoptries, in myoblast-derived *Tg*EVs compared to fibroblast- or Vero-derived *Tg*EVs. As well, in these TgEVs we observed a higher abundance of proteins related to nucleic acid-binding proteins suggesting a transfer of proteins involved in binding or interacting with DNA/RNA. The higher abundance of metal-binding proteins might impact enzymatic functions, or the structural stability of proteins and DNA delivered by EVs (Egli, 2002). This has led to the hypothesis that if myoblast may trigger spontaneous stage conversion of *T. gondii* (Ferreira-da-Silva et al., 2009; Rahman et al., 2021), then *T. gondii* EVs carry a different abundance cargo to prepare the transition. IPEC cells, or intestinal porcine epithelial cells, serve as a model for the intestinal epithelium studies in *C. suis*, another member within the Family Sarcocistidae (Worliczek et al., 2009). IPEC-1 are distinguished by their ability to produce cytokines and chemokines, which are essential components of innate immunity (Oswald, 2006). Their robust barrier functions and strong immune responses create a highly regulated environment that presents significant challenges for parasites like *T. gondii* to establish infection. When *T. gondii* infects IPEC-1 cells, it adapts its strategy to prioritize immune evasion and long-term survival rather than aggressive replication. This is likely influenced by the slower growth rate and lower metabolic activity of IPEC-1 cells, which have a doubling time of 40 hours and are generally in a non-proliferative state (Nossol et al., 2015). EVs released by *T. gondii* in IPEC cells reflect these adaptations, often carrying cargo that helps the parasite evade host immune responses, modulate stress pathways, and survive in nutrient-limited environments. In this context, *T. gondii* appears to adopt a survival-focused approach, avoiding the synthesis of proteins linked to rapid invasion or elevated replication. This may explain the lower abundance of invasion-related as micronemes and dense-granule proteins, DNA repair and ribosomal proteins in TgEVs derived. By contrast, fibroblast TgEVs are enriched with invasion-related proteins, highlighting a distinct strategy in these cells, where *T. gondii* enhances its invasive capabilities to facilitate infection spread.

## 5 Conclusions

Our findings demonstrate that *T. gondii* adjust the number of EVs and the protein composition of its EVs in response to the cellular environment, revealing a conserved core of 1,244 proteins alongside host cell-specific variations. The ability of *T. gondii* to fine-tune its EVs underscores its evolutionary success as a pathogen capable of infecting a broad range of hosts with differing susceptibilities. These findings not only expand our understanding of the biological role of EVs in parasitic infections but also highlight their potential as biomarkers for host-specific infection states. Furthermore, the identification of distinct EV protein profiles across host cell types opens new avenues for therapeutic exploration, particularly in targeting EV-mediated pathways to disrupt parasite survival and dissemination.

## Data Availability

The datasets presented in this study can be found in online repositories. The names of the repository/repositories and accession number(s) can be found in the article/**Supplementary Material**.

## Supplementary material

Supplementary Figure 1 Workflow for the isolation and purification of *T. gondii*-derived EVs. Created with Biorender.

Supplementary Figure 2 Transmission electron microscopy images of TgEVs and tachyzoites of *T. gondii* grown in **(A)** myoblast, **(B)** Vero cells, and **(C)** IPEC cells. Scale bar: 100nm.

Supplementary Figure 3 Venn diagram comparing the *T. gondii* EV proteome identified in this study (TgEVs, red circle) with the proteomes reported by Ramírez-Flores et al. (blue circle) and Wowk et al. (yellow circle). The numbers in each region indicate the count of proteins uniquely detected (single‐circle areas) or shared (overlapping areas) across the three datasets.

Supplementary Table 1 Protein report.

Supplementary Table 2 Summary of differential expression of proteins. They are listed along with their annotation number in ToxoDB, protein name, biological function, and abundance (LogFC), in each comparison.

Supplementary Table 3 List of proteins related to invasion process. They are listed along with their annotation number in ToxoDB, protein name, biological function, and abundance (LogFC), in each comparison.
