# Supplementary figures and images for: The proteomic landscape of Toxoplasma gondii extracellular vesicles across diverse host cell types
Source: Front Cell Infect Microbiol. 2025 Mar 18;15:1565684. doi: 10.3389/fcimb.2025.1565684 (PMC11958994; doi:10.3389/fcimb.2025.1565684)

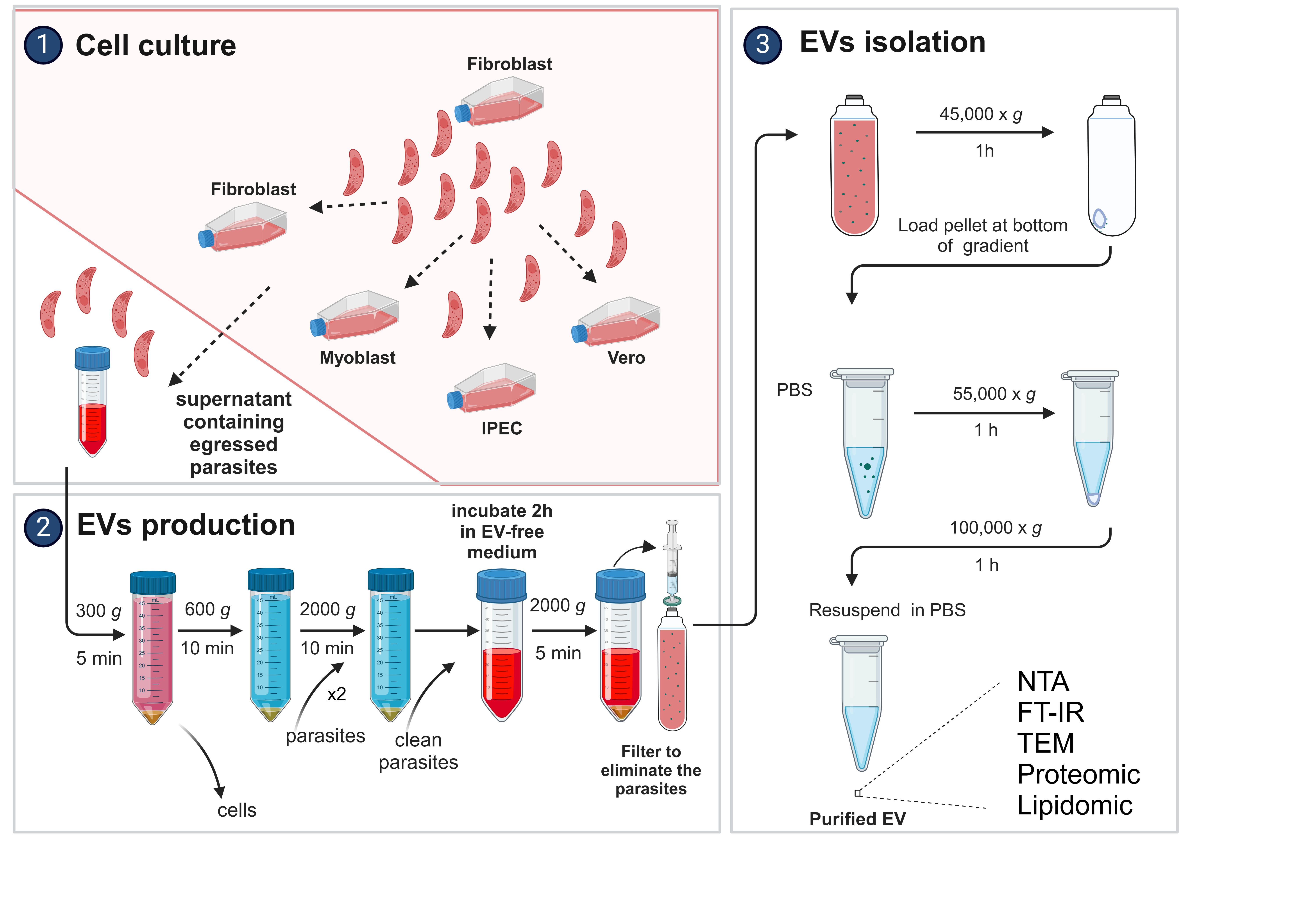

Supplement: Supplementary Figure 1 — Workflow for the isolation and purification of T. gondii-derived EVs. Created with Biorender. [file Image1.tif]

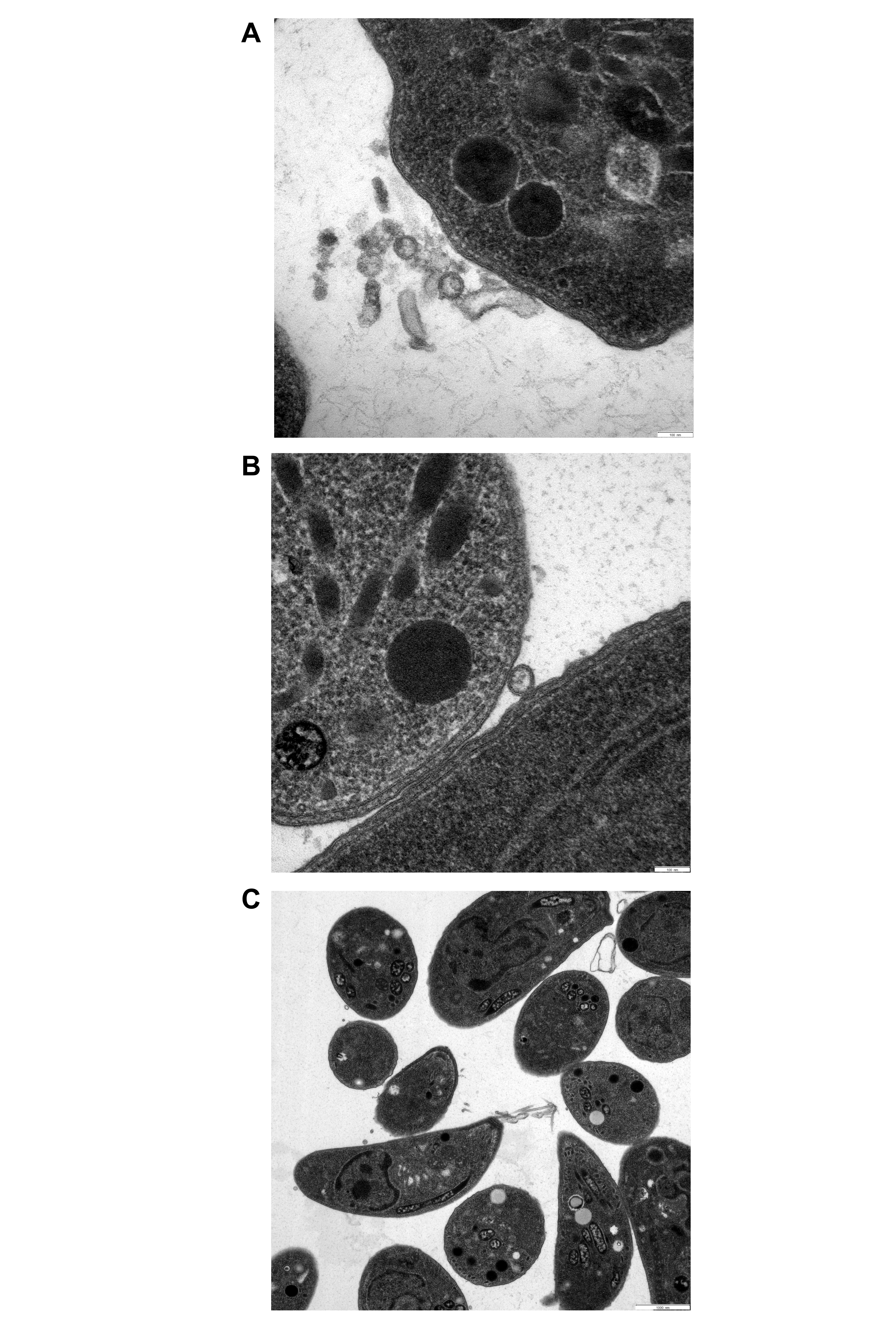

Supplement: Supplementary Figure 2 — Transmission electron microscopy images of TgEVs and tachyzoites of T. gondii grown in (A) myoblast, (B) Vero cells, and (C) IPEC cells. Scale bar: 100nm. [file Image2.tif]

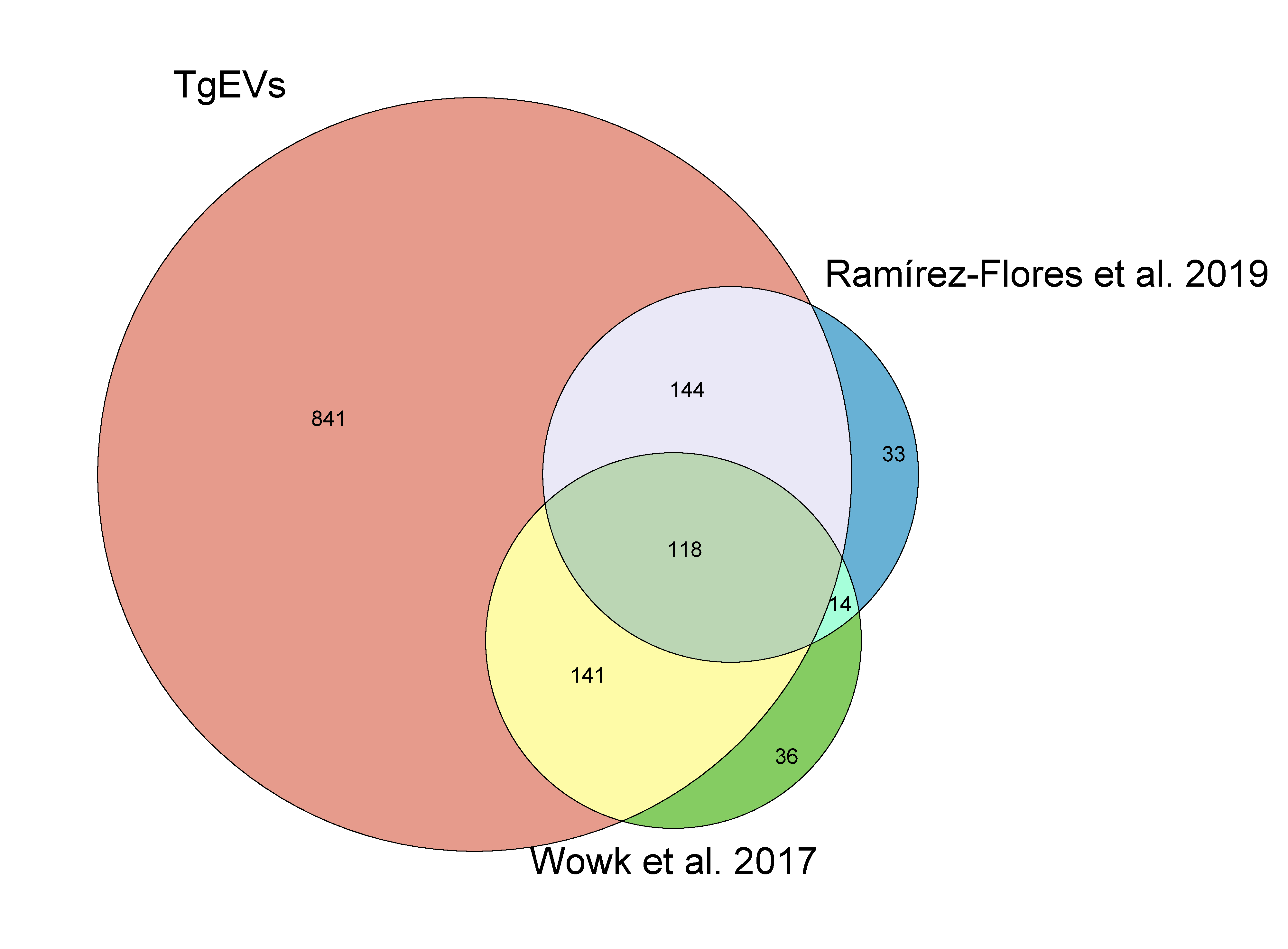

Supplement: Supplementary Figure 3 — Venn diagram comparing the T. gondii EV proteome identified in this study (TgEVs, red circle) with the proteomes reported by Ramírez-Flores et al. (blue circle) and Wowk et al. (yellow circle). The numbers in each region indicate the count of proteins uniquely detected (single‐circle areas) or shared (overlapping areas) across the three datasets. [file Image3.tif]
